# A Portable UV-LED Dual-Nafion Microfluidic Platform for Label-Free Trace Protein Preconcentration and Absorbance Detection

**DOI:** 10.3390/bios16100591

**Published:** 2026-10-21

**Authors:** Yi-Jia Shih, Yu-Sheng Chen, Chun-Ping Jen

**Affiliations:** 1 State Key Laboratory of Mariculture Breeding, Key Laboratory of Marine Biotechnology of Fujian Province, College of Marine Sciences, Fujian Agriculture and Forestry University, Fuzhou 350002, China; 2 Pingtan Research Institute, Xiamen University, Pingtan 350400, China; 3 Department of Mechanical Engineering and Advanced Institute of Manufacturing for High-Tech Innovations, National Chung Cheng University, Chiayi 62102, Taiwan; 4 School of Dentistry, College of Dental Medicine, Kaohsiung Medical University, Kaohsiung 80708, Taiwan

**Keywords:** UV-LED, microfluidic preconcentration, ion concentration polarization, dual-Nafion membrane, label-free UV absorbance, protein detection, portable analytical platform

## Abstract

Sensitive label-free detection of low-abundance proteins requires both effective on-chip preconcentration and a compact, low-noise optical readout for use outside conventional laboratories. Here, we present a portable UV-LED dual-Nafion microfluidic platform that integrates a 280 nm UV-LED, a chromium-coated slot collimator, a dual-Nafion ion concentration polarization (ICP) preconcentrator, and a miniature spectrometer within a 3D-printed enclosure. The symmetric dual-Nafion geometry on a UV-transparent quartz substrate confines the ICP enrichment zone to a fixed 120 × 500 µm interrogation window, enabling spatially stable, label-free on-chip absorbance monitoring at 280 nm. Using bovine serum albumin (BSA) as a model protein, the platform showed a linear absorbance response over 0–60 µM (R^2^ = 0.9933) and a static-loading limit of detection of 0.91 µM. Under a 30 V bias in 0.3 mM phosphate-buffered saline, representative 60 min experiments produced apparent enrichment factors of approximately 41, 4.5 × 10^3^, and 1.6 × 10^4^ at initial BSA concentrations of 1 µM, 10 nM, and 1 nM, respectively. At 10 pM, a reproducible, voltage-dependent absorbance increase was detected with three independent chips, whereas the 0 V and BSA-free controls remained below the detection threshold. The platform provides a practical approach for label-free trace-protein analysis and a potential upstream module for biomarker-specific assays.

## 1. Introduction

Sensitive detection of low-abundance proteins is important for clinical diagnostics, environmental monitoring, food safety analysis, and aquaculture pathogen surveillance. Many disease- and pathogen-relevant protein biomarkers occur in biological and environmental samples at nanomolar to sub-picomolar concentrations. Serum cytokines such as interleukin-6 (IL-6) and tumor necrosis factor-α (TNF-α) may occur at femtomolar to picomolar levels [1], whereas cardiac and tumor markers, including cardiac troponin I and prostate-specific antigen, are reported at picomolar to nanomolar concentrations [2]. Viral envelope and capsid proteins associated with aquaculture pathogens may likewise be present at low concentrations during early infection stages [3]. These concentration ranges can challenge compact analytical methods intended for point-of-need use [4,5]. In aquaculture and other resource-limited settings, there is a need for compact, low-cost analytical platforms capable of enriching dilute protein targets before downstream detection [6,7]. Enzyme-linked immunosorbent assay (ELISA) remains a widely used method for protein quantification; however, its multi-step sample preparation, long incubation times, and reliance on labeled secondary antibodies can limit rapid implementation outside centralized laboratories [8]. Mass spectrometry provides high sensitivity and molecular specificity but requires sophisticated instrumentation, skilled operators, and substantial operational resources [9,10]. Electrochemical biosensors offer miniaturizable, low-cost alternatives compatible with portable readout electronics [11,12]; however, their response at sub-nanomolar analyte concentrations may remain limited without an upstream enrichment step. Together, these considerations motivate integrated, compact analytical platforms that combine on-chip analyte preconcentration with low-noise optical readout.

Microfluidic preconcentration is an effective approach for addressing the mismatch between low bulk analyte concentrations and the detection limits of compact analytical systems [13]. Major electrokinetic preconcentration approaches include isotachophoresis (ITP) [14], isoelectric focusing (IEF) [15], field-amplified sample stacking [16] and ion concentration polarization (ICP). Solid-phase extraction has also been adapted for on-chip sample enrichment [17]. Among these approaches, ICP generated by nanoporous ion-selective membranes is particularly attractive for protein preconcentration because it can provide substantial enrichment under relatively simple buffer conditions without chemical modification of the target and can be coupled with either labeled or label-free optical readouts [18,19,20]. In ICP, a cation- or anion-selective membrane placed across a microchannel produces asymmetric ion transport under an applied DC electric field. This ion-selective transport generates ion-depleted and ion-enriched regions and can create a locally enhanced electric field that accumulates charged analytes into a concentrated zone [21]. Nafion, a sulfonated tetrafluoroethylene copolymer, has been widely used as a cation-selective material in ICP-based microfluidic preconcentrators because of its ion selectivity, chemical stability, and compatibility with solution-based microfabrication [22,23]. These characteristics make Nafion-based ICP a suitable upstream preconcentration strategy for label-free protein analysis, provided that the enrichment zone can be spatially confined for stable fixed-window optical interrogation.

Despite the substantial enrichment factors reported for single-Nafion membrane configurations [24,25], spatial instability of the enrichment zone remains a limitation for quantitative fixed-window detection. Under sustained DC bias, the concentrated band may migrate toward the membrane surface, broaden, or drift in response to evolving ionic gradients and electroosmotic flow, thereby complicating optical interrogation through a fixed measurement aperture [26]. Symmetric dual-Nafion architectures can confine the enrichment zone to a defined location between two ion-selective membranes and thereby provide the spatial stability needed for fixed-window optical monitoring [27]. In our previous work, a dual-Nafion quartz microfluidic chip incorporating a chromium-defined optical window enabled stable, label-free on-chip UV absorbance monitoring of electrokinetically enriched BSA under laboratory-based optical measurement conditions [27]. Building on that device-level demonstration, the present study focuses on system-level integration of the dual-Nafion preconcentrator with a compact optical readout. The platform combines a wavelength-targeted 280 nm UV-LED, a miniature spectrometer, and a 3D-printed optical enclosure to enable self-contained, fixed-window monitoring of label-free protein absorbance without a conventional optical table.

A further limitation of many ICP-based preconcentration systems is their reliance on fluorescence readout. Although fluorescence microscopy provides sensitive visualization of enrichment behavior, extrinsic labeling introduces additional sample-preparation steps and may alter protein conformation or electrophoretic mobility [28,29]. In contrast, UV absorbance at 280 nm enables label-free monitoring of proteins through the intrinsic absorption of aromatic residues, particularly tryptophan and tyrosine [30]. For proteins with sufficient absorbance at this wavelength, this approach can support reagent-free analysis of native proteins when the microfluidic device is coupled with a stable deep-UV optical path and a sufficiently sensitive readout. Conventional deuterium lamps provide broadband UV–visible illumination; however, their size, power consumption, heat generation, and optical-alignment requirements can complicate integration into compact microfluidic instruments. Deep-UV light-emitting diodes (UV-LEDs) emitting near 280 nm offer compact solid-state illumination with wavelength-targeted output, low power consumption, reduced heat generation, and long operational lifetime [31,32,33]. In the present platform, the UV-LED does not alter the underlying ICP enrichment mechanism; rather, its compact form factor and wavelength-targeted output facilitate co-integration of the optical source, dual-Nafion quartz chip, and miniature spectrometer within a 3D-printed enclosure for fixed-window, label-free monitoring of absorbance at 280 nm ($A_{280}$).

Building on our previously reported dual-Nafion quartz microfluidic preconcentrator with integrated label-free UV absorbance detection [27], the present work develops a portable analytical platform in which the chip is co-integrated with a 280 nm UV-LED, a chromium-coated slot collimator, and a microspectrometer within a 3D-printed enclosure. The dual-Nafion chip architecture and its ICP-based spatial-confinement mechanism were retained from the preceding device-level study; the principal advance of the present work is the integration of a compact, wavelength-targeted UV source and miniaturized optical readout into a self-contained platform that does not require a conventional optical table. Bovine serum albumin (BSA) was used as a model protein to evaluate on-chip absorbance calibration and electrokinetic preconcentration under controlled buffer conditions. The on-chip $A_{280}$ response was first calibrated over the 0–60 µM range to establish the calibration relationship used to convert fixed-window absorbance responses into calibration-derived apparent local BSA concentrations. Time-dependent enrichment was evaluated for initial BSA concentrations from 1 µM to 10 pM. The apparent enrichment factors obtained with the UV-LED were also compared with those obtained previously with a deuterium-lamp system using the same chip design [27] to examine whether replacing the benchtop broadband source with the compact UV-LED affects the measured enrichment. This comparison does not imply that the UV-LED changes the underlying ICP-driven transport process, which is governed by the applied electric field, membrane geometry, buffer composition, and analyte transport properties. Finally, the performance of the integrated platform was benchmarked against representative microfluidic preconcentration systems, including ICP/Nafion membrane devices, isotachophoresis-based systems, and nanofluidic electrokinetic trapping methods. The present work therefore demonstrates a compact, fixed-window dual-Nafion ICP and UV-LED absorbance platform for label-free trace-protein analysis; validation with target-specific assays and complex sample matrices will be required for translation to biomarker applications.

## 2. Device Design and Operating Principle

The proposed platform integrates four principal components: a PDMS/quartz microfluidic chip incorporating dual Nafion membranes, a UV-LED light source operating in the 270–280 nm range, a chromium-coated slot collimator, and a compact microspectrometer housed within a 3D-printed enclosure. The following subsections describe the chip architecture, the electrokinetic operating principle underlying protein preconcentration, and the UV absorbance detection scheme.

### 2.1. Chip Architecture and Dual-Nafion Configuration

**Figure 1:**
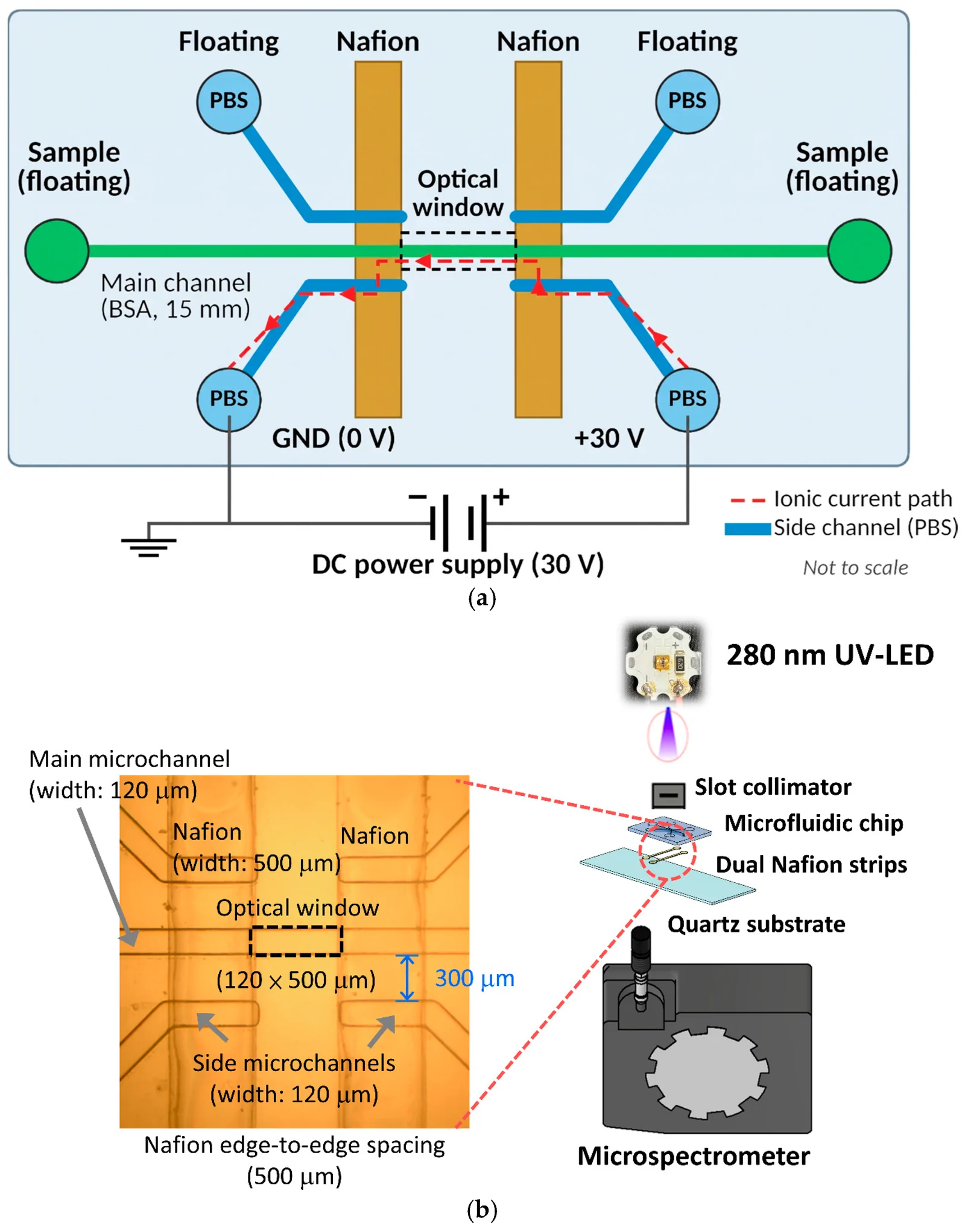
Design, electrode configuration, and optical integration of the dual-Nafion microfluidic platform. (**a**) Schematic layout of the chip (not to scale), showing the 15 mm main microchannel with the two sample reservoirs, the two Nafion strips, the four side microchannels with their PBS-filled reservoirs, and the optical window (dashed black box) between the Nafion strips. The lower-right side-channel reservoir was held at +30 V, and the lower-left side-channel reservoir was grounded (0 V) through platinum wire electrodes connected to a DC power supply; the two sample reservoirs and the two upper side-channel reservoirs were left floating. The dashed red line indicates the ionic current path through the right Nafion strip, the inter-membrane segment of the main microchannel, and the left Nafion strip. (**b**) (**Left**): Optical micrograph of the central chip region, showing the 120 µm-wide main microchannel, 120 µm-wide side microchannels, two 500 µm-wide Nafion strips, and the 120 × 500 µm optical window located between the Nafion strips. The Nafion edge-to-edge spacing is 500 µm, and the wall-to-wall gap between the main microchannel and each adjacent side microchannel is 300 µm. (**Right**): Exploded schematic of the portable optical module comprising a 280 nm UV-LED, slot collimator, dual-Nafion PDMS/quartz microfluidic chip, and microspectrometer.

The microfluidic chip consists of a polydimethylsiloxane (PDMS) layer bonded to a UV-transparent quartz substrate. The PDMS channel network comprises one 15 mm-long main microchannel connecting two sample reservoirs and four side microchannels, each connected to a separate reservoir, as shown in Figure 1. All channels are 120 µm wide and 40 µm high. Each side microchannel terminates over one of the Nafion strips described below, running parallel to the main microchannel and separated from its outer wall by a 300 µm wall-to-wall gap; the side microchannels are therefore electrically connected to the main microchannel only through the Nafion strips. Two Nafion strips, each 500 µm wide, are patterned on the quartz substrate perpendicular to the main microchannel, with an edge-to-edge spacing of 500 µm. The region between the two Nafion strips defines a 120 × 500 µm optical interrogation window that also contains the ICP enrichment zone during operation.

A chromium-coated slot collimator with a 120 µm-wide and 500 µm-long aperture is affixed to the top surface of the chip above the optical interrogation window. The aperture dimensions match the 120 µm main-channel width and the 500 µm edge-to-edge spacing between the Nafion strips, thereby defining the fixed 120 × 500 µm optical interrogation area. This configuration restricts the incident UV illumination primarily to the inter-Nafion region and reduces irradiation outside the active channel area. The collimator design was adapted from a previously reported single-Nafion fluorescence platform [34], which used a 120 µm × 700 µm slot aperture; the shorter aperture length used here matches the dual-Nafion confinement region. Quartz was selected as the substrate because of its deep-UV transmittance; in contrast, conventional soda-lime glass strongly absorbs below approximately 320 nm and is unsuitable for absorbance measurements at 280 nm [27].

### 2.2. ICP Operating Principle and Enrichment Zone Stabilization

Protein preconcentration is achieved through ion concentration polarization (ICP) at the Nafion membrane interfaces [18,21,22]. The Nafion strips act as cation-selective membranes. Under an applied DC electric field, cations preferentially pass through the Nafion while anions are largely excluded, creating ion-depleted and ion-enriched regions on opposite sides of each membrane. The resulting ion-concentration gradient produces a locally enhanced electric field within the depleted region. Electroosmotic flow transports the protein-containing solution toward the Nafion strips, whereas negatively charged protein molecules experience electrophoretic migration in the opposite direction. The balance between electroosmotic transport and electrophoretic migration causes proteins to accumulate near the membrane interfaces [27,34].

In single-Nafion devices, the enrichment zone can migrate axially along the main microchannel during sustained DC operation because of the asymmetric electrokinetic conditions associated with a single membrane [22,26]. Such axial drift can reduce measurement reproducibility and complicate optical monitoring through a fixed interrogation window. In the present device, two Nafion strips are arranged symmetrically on opposite sides of the optical interrogation window to confine the enrichment zone within the inter-membrane region. Under the applied bias, the ion-depletion regions generated at the two Nafion strips extend toward the central region, producing a confined zone in which electroosmotic transport, electrophoretic migration, and diffusion are balanced. As a result, the enrichment zone remains spatially localized near the inter-membrane region rather than propagating along the main channel [27]. This spatial confinement enables time-resolved absorbance monitoring through the fixed collimator aperture.

### 2.3. UV-LED Optical Detection Scheme

Protein absorbance near 280 nm arises primarily from aromatic amino acid residues, including tryptophan and tyrosine, and can be used for label-free optical monitoring without exogenous fluorescent labels [30]. For the quantitative absorbance measurements in this work, $A_{280}$ detection avoids fluorescent-dye conjugation steps and their associated sources of sample-preparation variability, potential perturbation of native proteins, and photobleaching [28,29].

**Figure 2:**
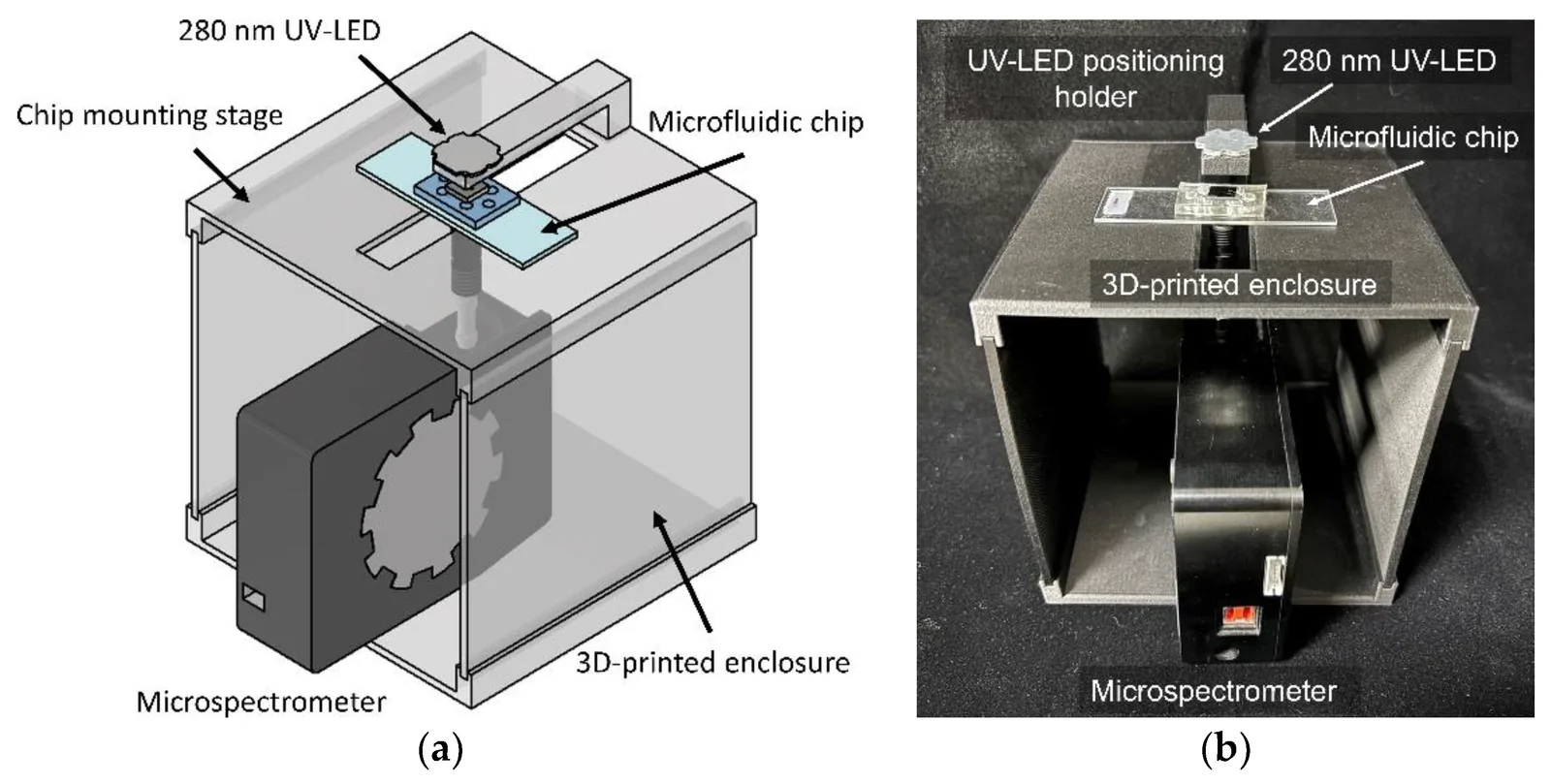
Compact 3D-printed enclosure (130 mm × 130 mm × 130 mm, length ×width × height) for UV absorbance monitoring. (**a**) Three-dimensional CAD schematic showing the 280 nm UV-LED, chip mounting stage, microfluidic chip, microspectrometer, and 3D-printed enclosure. (**b**) Photograph of the fabricated enclosure with the UV-LED positioning holder, 280 nm UV-LED, microfluidic chip, and microspectrometer integrated in fixed alignment. The enclosure eliminates the need for an optical table and supports compact, label-free 280 nm absorbance monitoring of ICP-preconcentrated proteins.

In the present platform, a solid-state UV-LED with peak emission near 280 nm serves as the illumination source, and the UV-LED, microfluidic chip, and microspectrometer are integrated in fixed alignment within a compact 3D-printed enclosure (Figure 2). UV light is directed through the slot collimator onto the fixed optical interrogation window, and the transmitted light is collected by the microspectrometer for real-time absorbance calculation; the optical components and the absorbance calculation are described in Section 3.3 and Section 3.5, respectively. Compared with a conventional broadband deuterium lamp, a UV-LED provides a compact solid-state light source with low power consumption, reduced heat generation, and long operational lifetime, which facilitate integration within the 3D-printed enclosure. This detection strategy adapts the microspectrometer-based architecture established in the predecessor platform [34] for deep-UV label-free operation by using a UV-LED and a quartz-based dual-Nafion chip [27].

## 3. Materials and Methods

### 3.1. Materials and Reagents

Bovine serum albumin (BSA; ≥98% purity; molecular weight ~66.5 kDa; Sigma-Aldrich, St. Louis, MO, USA) was used as the model protein for label-free UV absorbance measurements. A BSA stock solution was prepared by dissolving lyophilized BSA in 0.3 mM phosphate-buffered saline (PBS, pH 7.4) and was diluted to working concentrations of 1 µM, 10 nM, 1 nM, and 10 pM before the experiments. Fresh working solutions were prepared for each measurement session. The same buffer composition was used for all enrichment experiments to maintain consistent ionic conditions [27]. A low-ionic-strength buffer was chosen because ICP-based preconcentration relies on the permselectivity of the Nafion membranes, which decreases with increasing ionic strength [21,22]; the use of 0.3 mM PBS also allowed direct comparison with our previously reported dual-Nafion device characterized under the same buffer conditions [27]. For qualitative fluorescence visualization of the enrichment-zone geometry, fluorescein isothiocyanate-labeled BSA (FITC-BSA; Sigma-Aldrich) was prepared at concentrations of 1 µM, 10 nM, and 1 nM in 0.3 mM PBS (pH 7.4). All aqueous solutions were prepared using deionized water with a resistivity of ≥18.2 MΩ·cm.

A Nafion perfluorosulfonic acid/PTFE copolymer dispersion (20 wt% in lower aliphatic alcohols and water; D2020, Chemours, Wilmington, DE, USA) was diluted with ethanol and deionized water to prepare a 10 wt% working dispersion for Nafion-strip deposition. Polydimethylsiloxane (PDMS; Sylgard 184, Dow Corning, Midland, MI, USA) was prepared by mixing the base and curing agent at a 10:1 mass ratio, followed by vacuum degassing before use.

### 3.2. Microchip Fabrication

Microfluidic chips were fabricated by soft lithography and oxygen-plasma bonding procedures adapted from previous reports [27,34]. A silicon master mold defining the channel network was fabricated by standard SU-8 photolithography. The PDMS prepolymer mixture was poured over the master mold, degassed under vacuum, and cured at 80 °C for 2 h. After curing, the PDMS layer was peeled from the mold, and six reservoir access ports (two sample reservoirs and four side-channel reservoirs; Figure 1a) were formed using a biopsy punch.

Two Nafion strips were patterned on a UV-transparent quartz substrate by the microflow patterning method [27,34]. A temporary PDMS guide channel (500 µm wide and 40 µm deep) was reversibly bonded to the quartz substrate at the intended location of each Nafion strip. Approximately 3 µL of the 10 wt% Nafion dispersion was introduced into the guide channel and drawn through by capillary action. Excess dispersion was removed by gentle negative pressure, and the substrate was heated at 95 °C for 10 min. After removal of the guide channel, a 500 µm-wide Nafion strip remained on the quartz substrate. The procedure was repeated to form a second Nafion strip with an edge-to-edge separation of 500 µm. The PDMS microchannel layer was then bonded to the Nafion-patterned quartz substrate by oxygen-plasma treatment, with the main microchannel aligned perpendicularly to and centered between the two Nafion strips.

The custom-designed slot collimator, with a 120 µm × 500 µm aperture, was fabricated on a thin quartz plate by chromium sputtering followed by photolithographic patterning. The collimator was aligned above the optical interrogation window and affixed to the chip before platform assembly.

### 3.3. Optical and Electrical Setup

The UV-LED, microfluidic chip, and microspectrometer were integrated within a compact 3D-printed enclosure, as shown in Figure 2, without the need for a conventional optical table. A UV-LED module (3535 UVC LED, Shenzhen Ruibaoguang Technology Co., Ltd., Shenzhen, China; peak emission near 280 nm) was mounted above the chip mounting stage. Transmitted UV light exiting the quartz substrate was collected by a microspectrometer (SE2030-025-FUV; OTO Photonic Inc., Hsinchu City, Taiwan). Spectral data were acquired in real time using a LabVIEW-based interface and transferred to a connected computer. The reference spectrum and baseline used for absorbance calculation are described in Section 3.4 and Section 3.5.

The electrode configuration is shown in Figure 1a. The two main-channel reservoirs at the ends of the 15 mm main microchannel were loaded with the BSA sample, and the four side-channel reservoirs were filled with 0.3 mM PBS. A constant DC voltage of 30 V was applied using a DC power supply (PS9611A, ABM, Hsinchu, Taiwan) through two platinum wire electrodes immersed in the PBS of the lower-right and lower-left side-channel reservoirs; the lower-right reservoir was held at +30 V, and the lower-left reservoir was grounded (0 V). No electrodes were placed in the two main-channel reservoirs or in the two upper side-channel reservoirs, which were left electrically floating. Because the floating reservoirs carried no current, the ionic current passed from the biased side channel through the right Nafion strip, along the inter-membrane segment of the main channel containing the optical window, and through the left Nafion strip to the grounded side channel. The applied potential was therefore dropped along this path, establishing ion concentration polarization at the Nafion interfaces and the local electric field that drives protein accumulation within the optical window. The voltage was applied continuously throughout the 60 min enrichment period. The same electrode configuration was used in our previous dual-Nafion study [27]. For the 0 V control experiments, the sample was loaded in the same manner, but no voltage was applied.

For comparison of enrichment performance, deuterium-lamp data for initial BSA concentrations of 1 µM, 10 nM, and 1 nM were taken from our previous study using the same dual-Nafion chip design [27]. In that study, a UV-visible deuterium-tungsten light source (LS-1, Ocean Optics Inc., Dunedin, FL, USA) was coupled to the chip through an optical fiber and a fiber-optic collimator, and the beam was confined by a slot collimator with the same 120 × 500 µm aperture. An additional experiment at 10 pM was performed using the same setup but was not included in [27]. The applied voltage (30 V) and buffer composition (0.3 mM PBS) were the same as those used for the UV-LED experiments. The light intensity and spectral bandwidth delivered to the detection window were not matched between the two optical systems.

### 3.4. Experimental Procedure

Before each experiment, the channels were rinsed with 0.3 mM PBS and allowed to equilibrate. A reference spectrum (I_0_) was recorded with the light source directed to the spectrometer without the chip and slot collimator in the optical path. The absorbance of each chip filled with PBS was then recorded and used as the baseline for that chip, as described in Section 3.5. For calibration measurements, BSA standards from 0 to 60 µM were introduced under zero-voltage conditions, and $A_{280}$ was measured using three independently fabricated chips at each concentration (*n* = 3). For enrichment experiments, BSA at the target initial concentration was loaded into both main-channel reservoirs (radius 2.5 mm; measured height 2.67 ± 0.21 mm), giving an initial loading volume of approximately 105 µL, and the voltage protocol was applied. To compensate for evaporation caused by UV irradiation and the applied voltage, 20 µL of the same sample was added to each reservoir three times during each experiment, resulting in a total sample volume of approximately 225 µL. Transmission spectra were acquired continuously for 60 min at all initial BSA concentrations. Representative single-chip time courses were collected for 1 µM, 10 nM, and 1 nM BSA. For the 10 pM condition, measurements at 30 V and the no-bias control at 0 V were each performed using three independently fabricated chips. PBS blank measurements at 30 V were also acquired using three independently fabricated chips under the same operating conditions. Spatial validation was performed using FITC-BSA under the same voltage protocol. Samples were imaged using an inverted fluorescence microscope to qualitatively assess enrichment-zone confinement.

### 3.5. Data Analysis

Absorbance at 280 nm was calculated from the recorded transmission spectra as

(1) $$A_{280}\left(t\right)=-\log_{10}\left[I_{280}(t)/I_{280,0}\right],$$

where $I_{280}(t)$ is the transmitted intensity at 280 nm recorded at time t during an enrichment experiment, and $I_{280,0}$ is the reference transmitted intensity at 280 nm recorded without the chip and slot collimator in the optical path (Section 3.4). Absorbance computed in this way includes the attenuation of the optical path itself, which arises mainly from the 120 × 500 µm aperture and the quartz and PDMS interfaces. Because absorbance is additive, this contribution was removed by subtracting the absorbance of the same chip filled with PBS for calibration measurements, or the initial reading of the same run for enrichment experiments (Equation (3)). Enrichment was quantified using a calibration-derived apparent enrichment factor, ${EF}_{app}$, defined as the ratio of the apparent local BSA concentration in the optical window at time t, $C_{app}(t)$, to the known initial bulk BSA concentration, $C_{0}$:

(2) $${EF}_{app}=C_{app}(t)/C_{0}$$

$C_{app}(t)$ was obtained from the change in $A_{280}$ relative to the initial reading of the same run, using the slope m of the BSA calibration curve established from the static-loading measurements described in Section 3.4:

(3) $$C_{app}\left(t\right)=C_{0}+\left[A_{280}\left(t\right)-A_{280}\left(0\right)\right]/m$$

Referencing each run to its own initial reading eliminates chip-to-chip variations in baseline absorbance. The calibration curve relates $A_{280}$ to BSA concentration over the experimentally validated 0–60 µM range. The apparent local BSA concentrations inferred for the 1 µM, 10 nM, and 1 nM conditions were within this calibration range; therefore, ${EF}_{app}$ values were obtained by interpolation rather than extrapolation. The term “apparent” indicates that ${EF}_{app}$ is inferred from the optical response through an external calibration rather than from a direct measurement of local BSA concentration. The symbol ${EF}_{app}$ is used for all enrichment factors obtained in this work, whereas “enrichment factor” is used for values reported in the literature. At 10 pM, the $A_{280}$ response could not be converted into ${EF}_{app}$ (see Section 4.3).

The static-loading limit of detection (LOD) of the UV absorbance module was estimated as

LOD = 3σ/m(4)

where σ is the standard deviation of the blank absorbance readings, and m is the slope of the BSA calibration curve. Regression analysis and data processing were performed using MATLAB (version R2023b).

## 4. Results and Discussion

The performance of the dual-Nafion UV-LED platform was evaluated through optical characterization, on-chip BSA calibration, time-dependent ICP preconcentration experiments, comparison with a deuterium-lamp optical system, and benchmarking against representative literature reports. The UV-LED emission spectrum was first characterized and compared with that of a deuterium lamp to assess the suitability of the UV-LED as a wavelength-targeted source for $A_{280}$ measurements. On-chip BSA calibration was then performed to establish the 0–60 µM calibration range used to convert fixed-window absorbance responses into calibration-derived apparent local BSA concentrations and ${EF}_{app}$ values. Time-dependent preconcentration of native BSA was evaluated at initial concentrations from 1 µM to 10 pM. The ${EF}_{app}$ values obtained with the UV-LED were compared with those obtained previously with a deuterium-lamp system using the same chip design and were further benchmarked against representative microfluidic protein-preconcentration systems reported in the literature. The practical scope and limitations of the present platform are discussed in the final subsection.

### 4.1. UV-LED Spectral Characterization and Optical Performance

**Figure 3:**
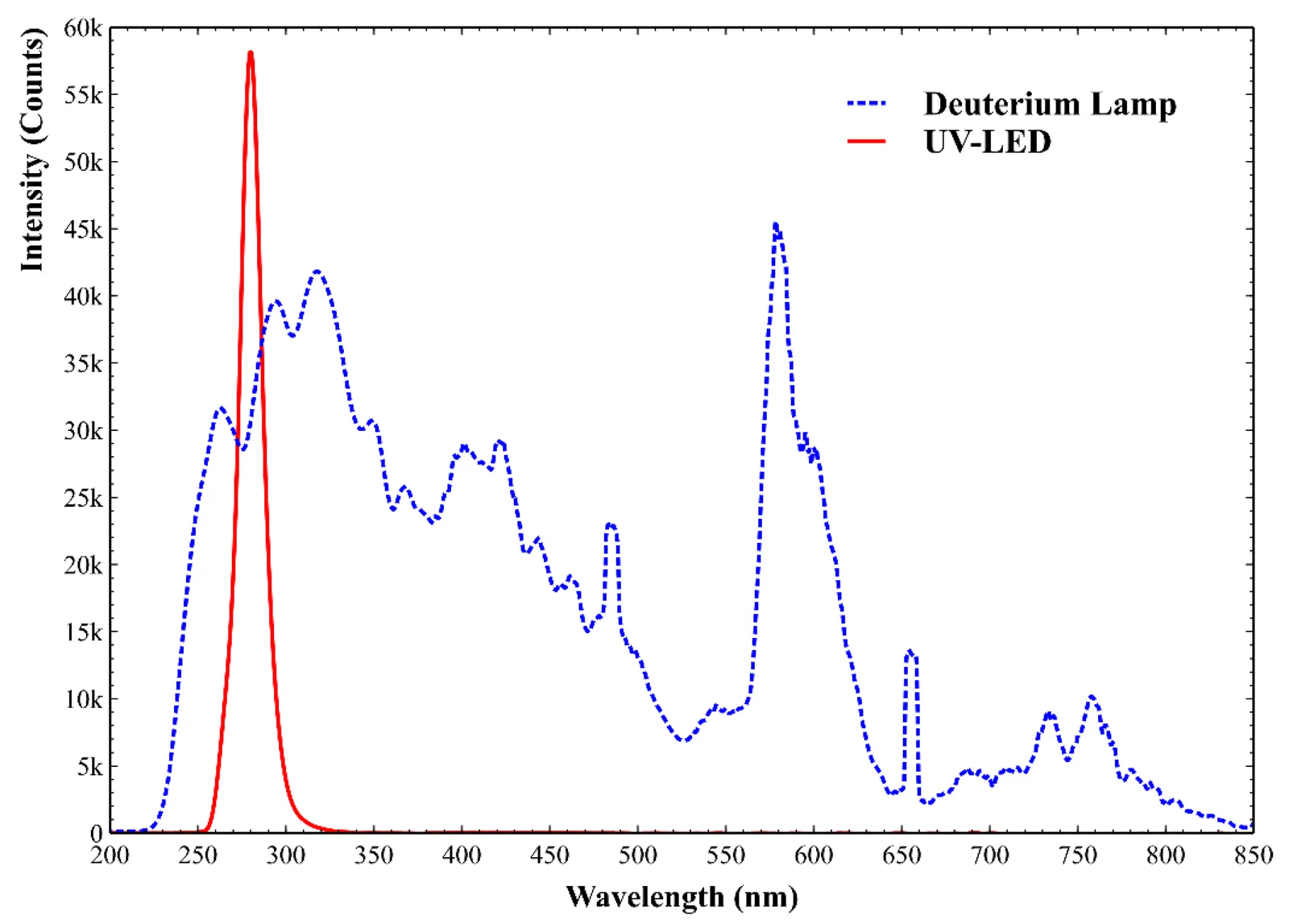
Emission spectra of the 280 nm UV-LED and deuterium lamp measured using the microspectrometer. The UV-LED (red solid line) exhibited a narrow emission band centered near 280 nm, whereas the deuterium lamp (blue dashed line) produced broadband emission across the measured spectral range of approximately 200–850 nm. The spectral overlap of the UV-LED output with the 280 nm protein-absorbance measurement wavelength supports its use as a compact, wavelength-targeted light source for label-free on-chip absorbance monitoring.

The emission spectra of the UV-LED and the deuterium-lamp system were measured using the microspectrometer and are shown in Figure 3. The UV-LED exhibited an emission band centered near 280 nm, which overlaps the wavelength used for protein absorbance monitoring [30]. In contrast, the deuterium lamp produced broadband emission across the measured spectral range of approximately 200–850 nm, with substantial intensity outside the $A_{280}$ measurement region. Thus, the UV-LED provides wavelength-targeted illumination for $A_{280}$ measurements, whereas the deuterium-lamp system provides broadband illumination over a wider spectral range.

The compact solid-state form factor of the UV-LED facilitates its integration with the microfluidic chip and microspectrometer within the 3D-printed enclosure. Unlike an arc-lamp source, the UV-LED does not require an arc-lamp warm-up procedure and can support rapid experimental startup [31]. Its wavelength-targeted emission and compact form factor are therefore advantageous for the present fixed-window, label-free $A_{280}$ absorbance-readout configuration.

### 4.2. On-Chip Calibration and Static-Loading Detection Limit

**Figure 4:**
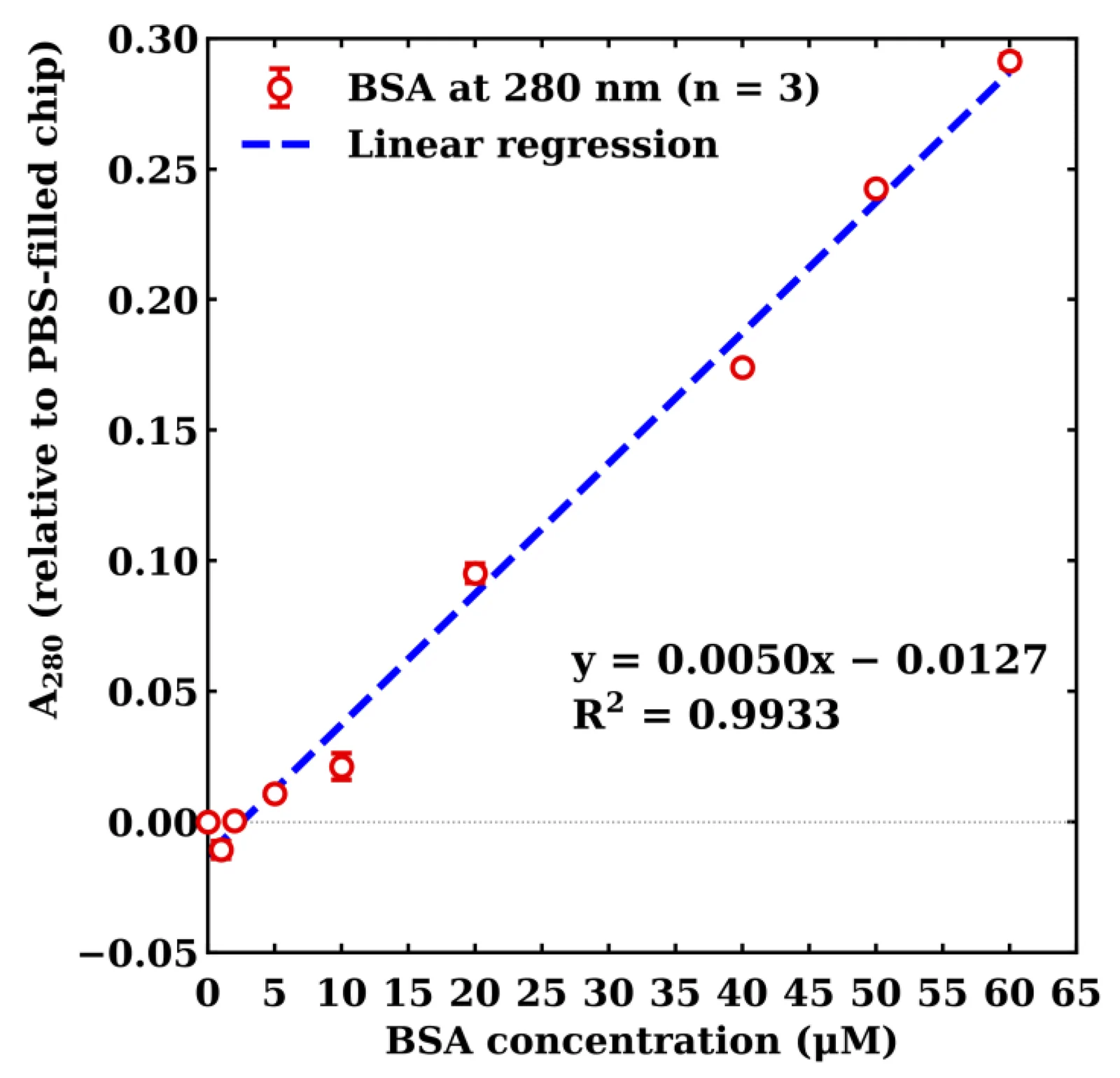
On-chip calibration of BSA absorbance at 280 nm using the UV-LED-integrated microfluidic platform. A_280_ is expressed relative to the PBS-filled chip (0 μM BSA in 0.3 mM PBS). Red open circles and error bars represent the mean ± standard deviation from three independently fabricated chips (*n* = 3). The blue dashed line denotes the linear regression fit, A_280_ = 0.0050C_BSA_ − 0.0127 (R^2^ = 0.9933), where A_280_ is the measured absorbance, and C_BSA_ is the BSA concentration in µM.

The relationship between fixed-window $A_{280}$ and BSA concentration was established by introducing BSA standards from 0 to 60 µM into the chip under zero-voltage conditions. The resulting calibration curve is shown in Figure 4, with $A_{280}$ expressed relative to the PBS-filled chip. A linear regression over the measured range gave A_280_ = 0.0050C_BSA_ − 0.0127, where C_BSA_ is the BSA concentration in µM, with R^2^ = 0.9933. At 60 µM, the BSA-dependent response (≈0.29) was approximately 190 times the standard deviation of the blank, confirming that the calibration reflects a protein-dependent signal well above the measurement noise. The calibration slope is, however, higher than that predicted from the Beer–Lambert law for a 40 µm optical path using the molar extinction coefficient of BSA (predicted slope ≈ 1.75 × 10^−4^ µM^−1^). The measured A_280_ is therefore regarded as an effective, system-specific optical response of the fixed-window configuration rather than a path-length-normalized molar absorbance. Possible contributions include the low transmitted intensity through the narrow aperture, which makes the measured intensity ratio sensitive to stray light and detector offset, light scattering, and protein adsorption at the channel surfaces. Because the calibration was obtained with the same chip design, optical configuration, and spectrometer settings used in the enrichment experiments, this system-specific response factor is incorporated in the calibration slope m, and no quantitative result in this work relies on the theoretical extinction coefficient. This calibration relationship was subsequently used to convert changes in fixed-window A_280_ responses during ICP experiments into calibration-derived apparent local BSA concentrations (Equation (3)). Minor deviations from the regression line were observed at the highest concentrations; however, all apparent local concentrations used for the ${EF}_{app}$ calculations were within the 0–60 µM calibration range.

Using the standard deviation of the blank absorbance (σ = 0.0015) and the slope of the calibration curve, the static-loading limit of detection of the UV absorbance module was estimated as LOD = 3σ/m ≈ 0.91 µM, where m is the calibration slope, and the limit of quantification as LOQ = 10σ/m ≈ 3.0 µM. These static-loading limits are higher than the nanomolar-to-picomolar initial BSA concentrations examined in the ICP experiments. However, ICP preconcentration increased the fixed-window $A_{280}$ response to values corresponding to apparent local BSA concentrations (approximately 41, 45, and 16 µM for initial concentrations of 1 µM, 10 nM, and 1 nM, respectively) above the LOQ and within the validated 0–60 µM calibration range. Accordingly, ${EF}_{app}$ values were determined by interpolation from the calibration curve rather than extrapolation beyond the calibrated concentration range. Because ${EF}_{app}$ is calculated from the change in A_280_ relative to the initial reading of each run (Equation (3)), it depends on the calibration slope but not on the calibration intercept.

### 4.3. Time-Dependent BSA Preconcentration Performance

Before label-free UV absorbance measurements, the spatial behavior of the enrichment zone under the applied voltage was qualitatively assessed by fluorescence imaging of FITC-labeled BSA at initial concentrations of 1 µM, 10 nM, and 1 nM. The fluorescence micrographs showed a concentrated protein band within the 500 µm inter-Nafion region that remained spatially localized between the two Nafion strips during 30 min of continuous operation. This observation is consistent with the intended dual-Nafion confinement design [18,21,27] and contrasts with the axial migration reported for single-membrane ICP devices under sustained bias [22,26]. Because FITC labeling can alter the charge and electrophoretic mobility of BSA, the fluorescence images were used only to qualitatively verify the location of the enrichment zone and not to quantify enrichment of native BSA. The quantitative results reported below were obtained from label-free fixed-window $A_{280}$ measurements.

**Figure 5:**
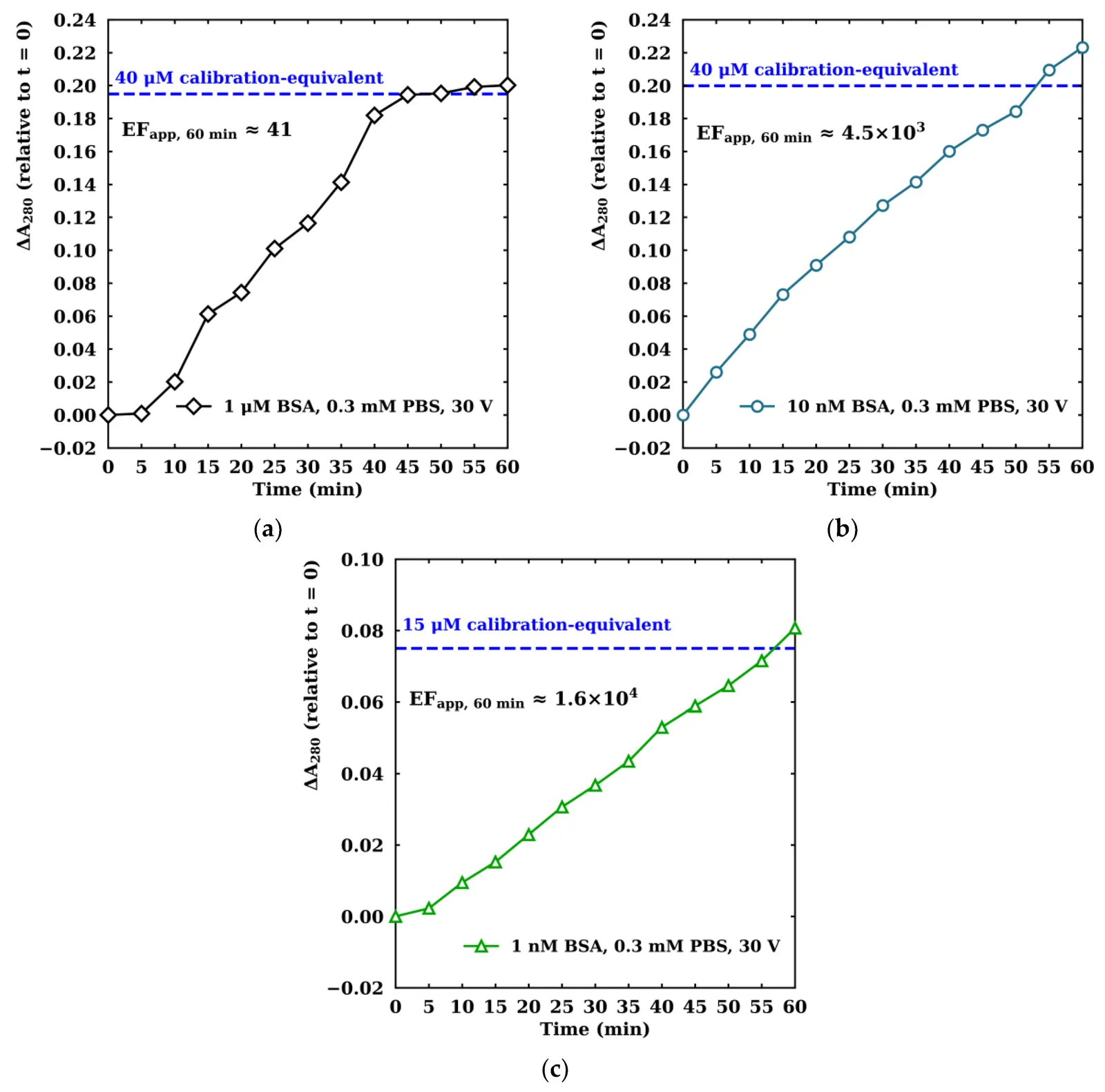
Representative time-dependent changes in fixed-window absorbance at 280 nm during dual-Nafion ICP preconcentration of BSA in 0.3 mM PBS at 30 V, monitored within the 120 × 500 µm optical window for 60 min. Initial BSA concentrations were (**a**) 1 µM, (**b**) 10 nM, and (**c**) 1 nM. ΔA_280_ is expressed relative to the same chip at t = 0. Dashed lines indicate the ΔA_280_ corresponding to the indicated calibration-equivalent BSA concentrations. EF_app_ values at 60 min were calculated using Equations (2) and (3), where C0 is the initial BSA concentration and m is the calibration slope (Figure 4). Note the different y-range in (**c**).

**Figure 6:**
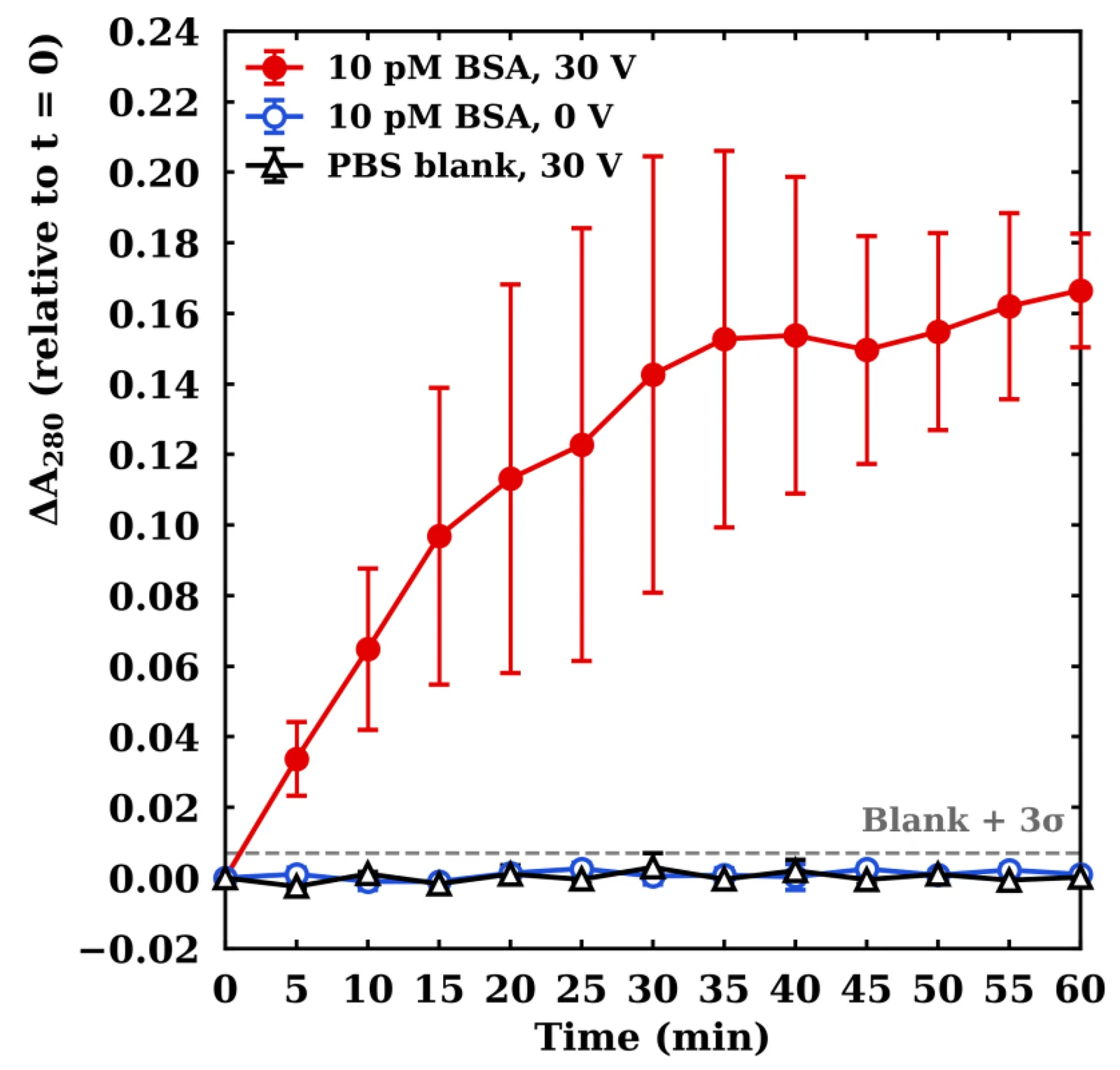
Time-dependent changes in fixed-window A_280_ for 10 pM BSA during dual-Nafion ICP preconcentration in 0.3 mM PBS. Red filled circles: 10 pM BSA at 30 V; blue open circles: 10 pM BSA at 0 V (no-bias control); black open triangles: PBS blank at 30 V. ΔA_280_ is expressed relative to the same chip at t = 0. Data are presented as mean ± standard deviation from three independently fabricated chips (*n* = 3). The dashed line indicates the detection threshold, defined as the mean plus three standard deviations of the ΔA_280_ values of the 30 V PBS blank over 5–60 min. Because the inferred amount of BSA would exceed the amount supplied to the chip (Section 4.3), this response is reported as qualitative detection, and no enrichment factor is assigned.

The time-dependent fixed-window $A_{280}$ responses during electrokinetic preconcentration of native BSA are shown in Figure 5 and Figure 6. Figure 5 presents representative single-chip time courses for initial BSA concentrations of 1 µM, 10 nM, and 1 nM. Under a 30 V bias in 0.3 mM PBS, the $A_{280}$ response increased over the 60 min monitoring period, corresponding to ${EF}_{app}$ values of approximately 41, 4.5 × 10^3^, and 1.6 × 10^4^, respectively. Because these time courses were each obtained using a single chip, these ${EF}_{app}$ values should be regarded as representative values indicating the order of magnitude of enrichment, rather than as precise values with a defined run-to-run uncertainty. Figure 6 presents the 10 pM BSA response at 30 V, the corresponding 0 V BSA control, and a PBS blank measured at 30 V. Data are shown as mean ± standard deviation from three independently fabricated chips for each condition. The $A_{280}$ response increased progressively for 10 pM BSA at 30 V, whereas the 0 V BSA control and the 30 V PBS blank remained near the baseline throughout the 60 min monitoring period. At 60 min, the 30 V BSA response reached ${∆A}_{280}$ = 0.167 ± 0.016 (*n* = 3), well above the blank-derived detection threshold of 0.0069, whereas neither control exceeded this threshold.

To assess whether the calibration-derived values are physically consistent with the amount of protein supplied to the chip, a molecular mass balance was performed. The optical window (120 × 500 × 40 µm) has a volume $V_{w}$ of 2.4 nL, and the total sample volume $V_{s}$ supplied during each experiment was approximately 225 µL (Section 3.4). If all BSA molecules in the supplied sample were transported into the window, the maximum attainable local concentration would be $C_{max}=C_{0}V_{s}/V_{w}$, and the maximum attainable enrichment factor would be ${EF}_{max}=V_{s}/V_{w}\approx 9.4\times 10^{4}$, independent of $C_{0}$. For the 1 µM, 10 nM, and 1 nM conditions, the calibration-derived enrichment factors (41, 4.5 × 10^3^, and 1.6 × 10^4^) are below this limit, and the raw sample volumes required to supply the inferred amounts of BSA in the window (approximately 0.1, 11, and 39 µL, respectively) are smaller than the volume supplied. At 10 pM, however, the 225 µL sample contains only approximately 2.25 fmol of BSA, so that the local concentration could not exceed approximately 0.94 µM, whereas the observed ${∆A}_{280}$ corresponds to a calibration-equivalent concentration of approximately 33 µM. Because $A_{280}=-\log_{10}I/I_{0}$ represents the total optical extinction within the window, the response at 10 pM likely includes contributions in addition to molecular absorption, such as light scattering from protein locally accumulated in the ICP zone. The 10 pM result is therefore reported as reproducible, voltage-dependent, label-free qualitative detection, and the enrichment factor at this concentration is represented only by its mass-balance upper bound (EF ≤ 9.4 × 10^4^).

${EF}_{app}$ increased as the initial BSA concentration decreased across the tested range. This trend reflects the concentration-normalized metric used to express enrichment and the ability of the fixed-window system to generate measurable BSA-derived absorbance responses at progressively lower starting concentrations. The 10 pM control data further show that no measurable increase in fixed-window $A_{280}$ occurred for the BSA sample in the absence of an applied voltage during the 60 min monitoring period. Because ${EF}_{app}$ was calculated from fixed-window $A_{280}$ responses using an external BSA calibration, it should be interpreted as an apparent value rather than as direct measurements of local BSA concentration. The conversion assumes that the $A_{280}$ response per unit BSA concentration in the enrichment zone is the same as that obtained under static, zero-voltage calibration conditions. Within the ICP zone, however, local changes in ionic strength and pH, high local protein concentration, and possible conformational changes, aggregation, or wall adsorption may alter the optical response. These effects were not independently verified in the present study. Because aggregation, scattering, or adsorption would increase the measured extinction, ${EF}_{app}$ may overestimate the concentration of dissolved, native BSA. Several features of the data nevertheless support the use of the conversion within the reported range. First, ${EF}_{app}$ was obtained from the change in $A_{280}$ relative to t = 0 of the same run using only the calibration slope (Equation (3)). Second, the 0 V BSA controls and 30 V PBS blanks remained below the detection threshold throughout 60 min (Figure 6), indicating that voltage application and buffer changes alone did not produce a measurable A_280_ response. Third, all apparent concentrations were above the LOQ and within the calibrated range. Finally, the corresponding BSA quantities did not exceed the amount supplied to the device. The spatial localization observed using FITC-BSA and the fixed dual-Nafion geometry supports stable monitoring within the inter-membrane optical window [26,27].

### 4.4. UV-LED Versus Deuterium–Tungsten Illumination

**Figure 7:**
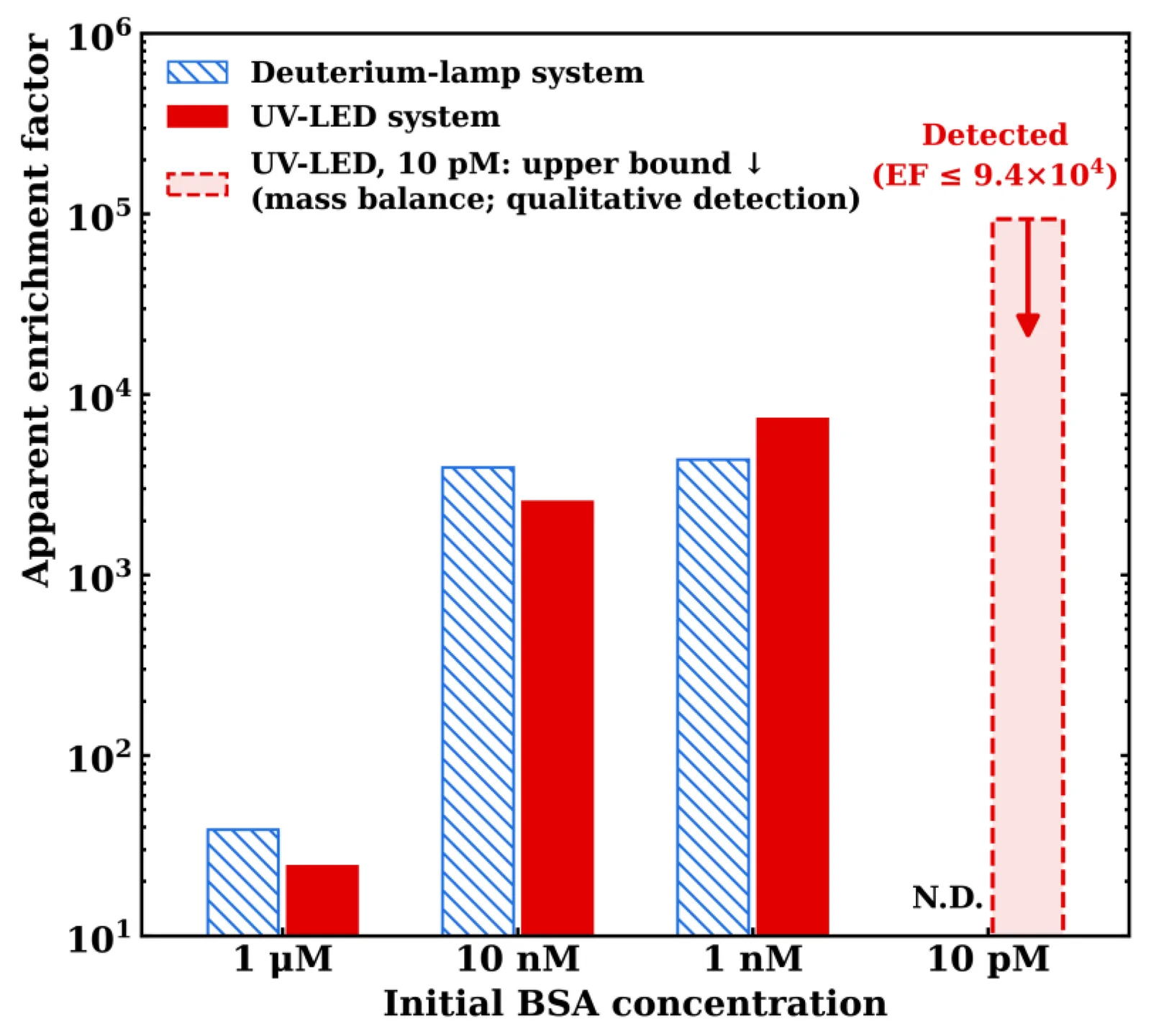
Comparison of apparent enrichment factors (EF_app_) obtained with the deuterium-lamp and UV-LED optical systems during dual-Nafion ICP preconcentration of BSA in 0.3 mM PBS at 30 V, for initial concentrations of 1 µM, 10 nM, 1 nM, and 10 pM, plotted on a logarithmic scale. Values were evaluated at a common enrichment time of 30 min. Deuterium-lamp values are those reported previously for the same dual-Nafion chip design [27]; UV-LED values were calculated from the change in A_280_ relative to the initial reading of each run using the UV-LED calibration slope (Figure 4). Values at 1 µM, 10 nM, and 1 nM are from representative single-chip runs. At 10 pM, the UV-LED system detected a reproducible, voltage-dependent A_280_ response (*n* = 3; Figure 6) that could not be quantified; the dashed bar with a downward arrow indicates the mass-balance upper bound of the enrichment factor (EF ≤ 9.4 × 10^4^; Section 4.3). N.D.: no response distinguishable from baseline fluctuation was observed with the deuterium-lamp system at 10 pM (additional experiment not included in [27]).

To examine whether replacing the benchtop broadband source with the compact UV-LED affects the measured enrichment, the ${EF}_{app}$ values obtained with the UV-LED were compared with the enrichment factors reported previously for the same dual-Nafion chip design operated with a deuterium–tungsten light source under the same applied voltage (30 V) and buffer composition (0.3 mM PBS) [27]. Because the deuterium-lamp values were reported at 30 min, the UV-LED values were evaluated at the same enrichment time (Figure 7). In both systems, enrichment was quantified using the on-chip calibration curve of the corresponding optical system; because absorbance is calculated from the ratio of transmitted to reference intensity recorded with the same source, the enrichment factor is largely insensitive to differences in source intensity. At 30 min, the UV-LED gave ${EF}_{app}$ values of 24, 2.5 × 10^3^, and 7.4 × 10^3^ at initial BSA concentrations of 1 µM, 10 nM, and 1 nM, respectively, compared with 39, 3960, and 4380 for the deuterium-lamp system [27]. These values agreed within a factor of two, and neither source gave consistently higher values. Because the values were obtained from single-chip experiments and the enrichment factors in [27] were calculated from the final absorbance rather than from the change relative to the initial reading, these differences are not interpreted further. This agreement is consistent with the enrichment being governed by the dual-Nafion chip and the applied electric field rather than by the illumination source.

At 10 pM, the UV-LED platform detected a reproducible, voltage-dependent $A_{280}$ response (*n* = 3; Figure 6), whereas no response distinguishable from baseline fluctuation was observed in an additional experiment performed with the deuterium-lamp setup. However, the two optical systems differed in spectral bandwidth (Figure 3), optical coupling, and the light intensity delivered to the detection window, and these parameters were not matched or normalized. Because light intensity and spectral bandwidth influence the baseline noise and stray-light level, and the two data sets were obtained in separate experimental series, the difference at 10 pM cannot be attributed specifically to the light source. The present comparison therefore shows that the compact UV-LED could replace the benchtop broadband source without loss of enrichment performance at the concentrations compared, rather than demonstrating source-dependent differences in detectability. A direct assessment of source-dependent detectability would require measurement of the light intensity and spectral bandwidth at the detection window, normalization of the transmitted intensity, and side-by-side replicate experiments with both sources. The comparison does not imply that the UV-LED changes the underlying ICP transport mechanism or directly alters the local protein concentration generated by the dual-Nafion device.

### 4.5. Contextual Benchmarking Against Reported Preconcentration Platforms

**Figure 8:**
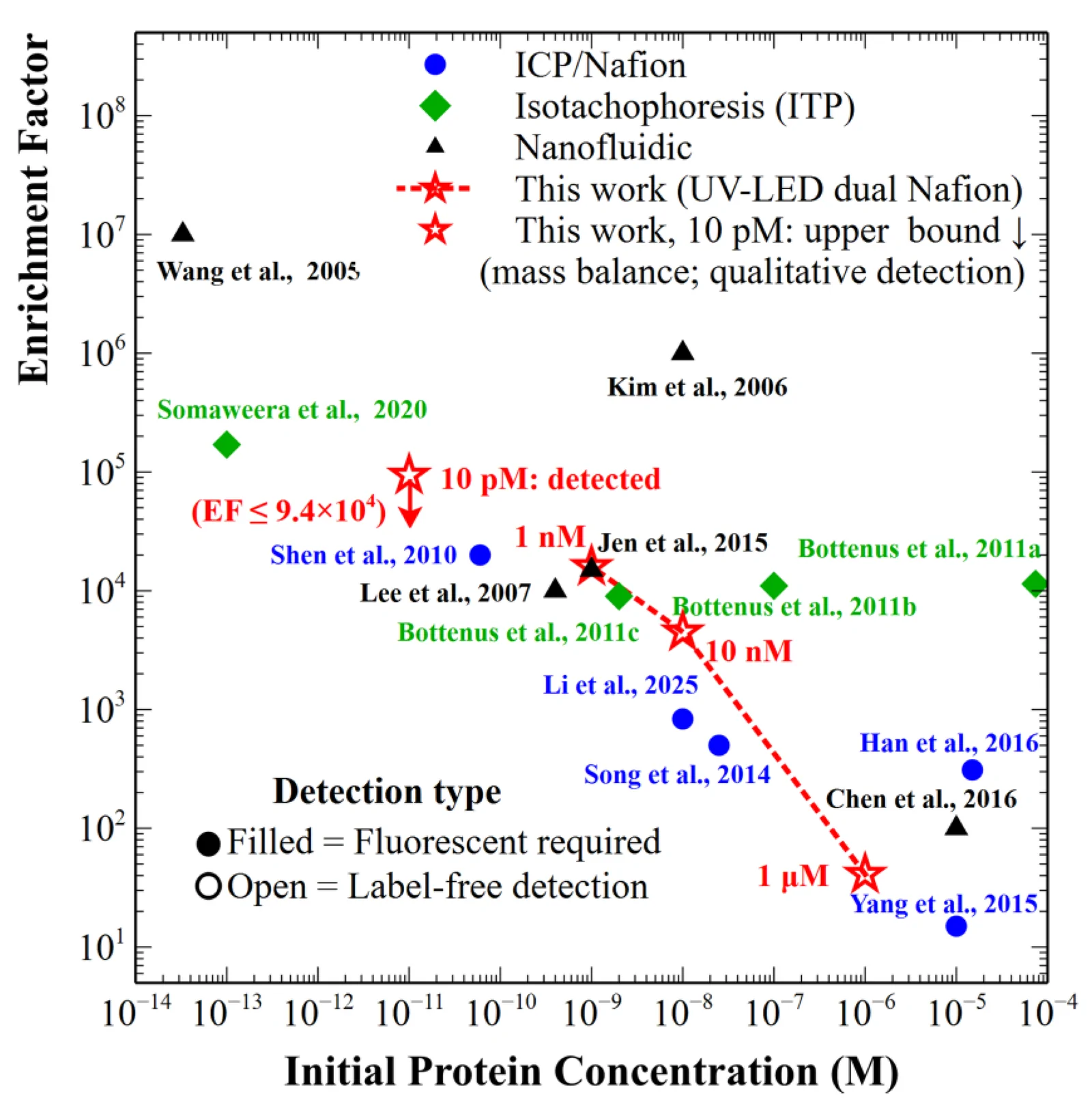
Comparison of enrichment factors versus initial protein concentration for representative microfluidic preconcentration systems for proteins and other charged analytes reported in the literature. Blue circles, green diamonds, and black triangles represent ion concentration polarization (ICP)/Nafion, isotachophoresis (ITP), and nanofluidic electrokinetic trapping systems, respectively. Filled and open markers denote detection requiring fluorescent labeling and label-free detection, respectively. Red open stars connected by the dashed line indicate the EFapp values obtained in this work after 60 min by label-free UV absorbance at 280 nm at initial BSA concentrations of 1 μM, 10 nM, and 1 nM. At 10 pM, a reproducible, voltage-dependent A_280_ response was detected but could not be quantified; the red star with a downward arrow denotes the upper bound of the enrichment factor (EF ≤ 9.4 × 10^4^), determined by mass balance from the total amount of BSA supplied to the chip (Section 4.3). Because the reported studies differ in analyte identity, sample matrix, ionic strength, device geometry, operating conditions, and the definition of enrichment factor, this figure is intended as a contextual comparison rather than a direct quantitative ranking. Data sources: ICP/Nafion—Shen et al., 2010 [22]; Song et al., 2014 [35]; Yang et al., 2015 [36]; Han et al., 2016 [23]; Li et al., 2025 [34]. ITP—Bottenus et al., 2011a–c [37,38,39]; Somaweera et al., 2020 [40]. Nanofluidic—Wang et al., 2005 [20]; Kim et al., 2006 [24]; Lee et al., 2007 [25]; Jen et al., 2015 [41]; Chen et al., 2016 [42].

Figure 8 places the present UV-LED dual-Nafion platform in the context of representative microfluidic preconcentration systems reported in the literature, including Nafion-based ion concentration polarization (ICP) devices, isotachophoresis (ITP)-based systems, and nanofluidic electrokinetic trapping platforms. Data points are categorized by preconcentration mechanism and by whether the reported optical readout required fluorescent labeling. Because the reported studies differ in analyte identity, sample matrix, ionic strength, device geometry, applied voltage, enrichment duration, and the method used to define or infer enrichment factor, Figure 8 is intended as a contextual comparison rather than a direct quantitative ranking.

Under the present operating conditions, the UV-LED dual-Nafion platform produced ${EF}_{app}$ values of approximately 4.5 × 10^3^ at 10 nM and 1.6 × 10^4^ at 1 nM BSA after 60 min. At 10 pM, the 30 V experiment produced a reproducible, time-dependent $A_{280}$ response above the blank-derived detection threshold, while the 0 V BSA control and the 30 V PBS blank remained below this threshold; this response is reported as qualitative detection, with the enrichment factor bounded by mass balance (EF ≤ 9.4 × 10^4^). These results demonstrate that the platform can generate measurable, label-free $A_{280}$ responses from initial BSA concentrations extending from the nanomolar range to 10 pM. Relative to the predecessor dual-Nafion quartz platform, which employed a benchtop deuterium light source, and the previously reported PDMS-based Nafion platform from this group, the present result shows that dual-Nafion confinement can be combined with compact UV-LED-based fixed-window absorbance monitoring of native BSA in a self-contained enclosure [27,34].

Representative ITP-based systems have reported enrichment factors on the order of 10^4^–10^5^ for proteins and other charged analytes, including anionic ITP focusing of protein mixtures with approximately 10^4^-fold enrichment [37], cationic ITP enrichment of cardiac troponin I [38], and ITP-based preconcentration and detection of phosphorylated cardiac troponin I isoforms [39]. Nanofluidic electrokinetic trapping systems constitute another category of high-performance preconcentrators and have reported enrichment factors on the order of 10^6^–10^7^ in selected configurations [20,25]. Electrokinetic protein preconcentration has also been demonstrated using glass–PDMS microfluidic devices [24]. Previous studies from this group likewise used nanofluidic confinement, including nanofracture junctions combined with gold-nanoparticle-enhanced detection and nano-interstice devices monitored using fluorescence readout [41,42,43]. Such platforms can generate strong enrichment; however, their performance is influenced by nanoscale-junction fabrication, electrolyte formulation, sample composition, operating parameters, and the detection configuration.

Within this literature context, the present device offers a distinct combination of solution-patterned Nafion films on quartz, dual-Nafion ICP confinement, a spatially fixed optical interrogation window, and label-free UV absorbance monitoring of native BSA within a compact 3D-printed enclosure. Rather than establishing a universal performance ranking across dissimilar preconcentration methods, Figure 8 highlights the practical niche of the present design: a compact and label-free platform that enables time-resolved absorbance monitoring of an ICP-confined protein zone from bulk BSA concentrations extending to 10 pM. Practical performance should therefore be considered not only in terms of enrichment factor, but also in terms of enrichment time, optical readout, labeling requirement, device-fabrication complexity, zone stability, and compatibility with the intended sample matrix.

### 4.6. Practical Considerations and Translational Potential

Integration of the UV-LED, chromium-coated slot collimator, dual-Nafion microfluidic chip, and micro-spectrometer within a single 3D-printed enclosure (130 mm × 130 mm × 130 mm, length ×width × height) eliminates the need for a conventional optical table and reduces the optical alignment burden associated with a benchtop arrangement. The compact configuration therefore provides a practical basis for portable or space-constrained laboratory use. As shown in Section 4.4, the ${EF}_{app}$ values obtained with the UV-LED were comparable to those obtained previously with a benchtop deuterium–tungsten source, indicating that the compact source could be adopted without loss of enrichment performance at the concentrations compared. In addition, absorbance near 280 nm can provide a label-free readout for proteins containing UV-absorbing aromatic residues, including tryptophan and tyrosine [30]. Nevertheless, the magnitude of the $A_{280}$ response depends on the protein-specific abundance and local environment of these residues, as well as on the optical path length and sample background. Consequently, the present 280 nm readout should be regarded as a general label-free protein-monitoring modality rather than an intrinsically target-specific assay. Target-specific quantification in mixtures would require an additional molecular-recognition or separation step.

Although BSA was used as a model protein in this study, ICP-based preconcentration is governed principally by analyte charge, electrophoretic mobility, electroosmotic transport, and the ionic composition of the sample solution [21,22]. The integrated device may therefore serve as a front-end preconcentration module for downstream biomarker-specific assays when the target protein has suitable electrokinetic properties, and the sample matrix is compatible with ICP operation, for example after dilution, desalting, or buffer exchange to reduce the ionic strength. Potential future applications include concentration of dilute protein biomarkers before affinity-based, electrochemical, or other selective readout steps in clinical, environmental, food-safety, and aquaculture-monitoring workflows [4,5,6,7]. However, direct use with complex samples has not been established in the present study. Validation will require systematic testing of matrix effects, ionic-strength tolerance, nonspecific adsorption, competing proteins, analyte recovery, and the performance of an integrated target-recognition element.

### 4.7. Study Limitations and Future Directions

The present study establishes the feasibility of integrating dual-Nafion ICP preconcentration with UV-LED-based, label-free absorbance monitoring; however, the results were obtained using purified BSA in 0.3 mM PBS and should be interpreted within this controlled model-system context. Biological and environmental samples can contain higher ionic strength, variable pH, surfactants, particulates, and competing macromolecules. In particular, the ionic strength of physiological fluids (approximately 150 mM) is more than two orders of magnitude higher than that of the buffer used here. At such ionic strength, the permselectivity of the Nafion membranes decreases, while the current and Joule heating increase, which would weaken the ion-depletion zone and reduce preconcentration efficiency; direct operation with undiluted blood, plasma, or urine is therefore not expected and has not been demonstrated. These factors can influence ionic transport and electrokinetic behavior in ion-selective micro- and nanofluidic systems [44] and may thereby affect ion concentration polarization, electroosmotic transport, protein mobility, and nonspecific adsorption in the present platform. Application to real samples would require dilution, desalting, or buffer exchange, which also dilutes the target analyte. For example, a 500-fold dilution of a physiological sample to the ionic strength used here would reduce an analyte present at approximately 5 nM to approximately 10 pM, the lowest initial concentration at which a voltage-dependent response was detected in this study. The ability to operate at very low initial concentrations is therefore a prerequisite for such workflows, although this estimate does not replace experimental validation. Future studies should therefore evaluate performance in relevant sample matrices and systematically assess the effects of ionic strength, pH, sample pretreatment, protein recovery, and matrix-derived optical background, including the identification of the maximum ionic strength compatible with effective ICP operation in the dual-Nafion device.

ICP-driven preconcentration is not inherently target-specific. Charged species with electrophoretic mobilities compatible with the operating conditions can co-accumulate in the inter-membrane zone, and the $A_{280}$ measurement reports the combined absorbance of UV-absorbing species within the fixed optical window. In complex samples, nucleic acids, small aromatic molecules, and non-target proteins may therefore contribute to the measured response, and their co-accumulation could drive the window response beyond the validated calibration range (0–60 µM BSA equivalent); in such cases, the enrichment time would need to be shortened or interfering species removed by sample pretreatment. The narrow emission band of the UV-LED (approximately 265–300 nm; Figure 3) concentrates the source intensity at the protein absorbance wavelength but precludes multi-wavelength discrimination with a single source. Because the microspectrometer records the full spectrum, such discrimination could be introduced by integrating a second LED into the same enclosure; for example, a 260 nm LED would allow the $A_{260}/A_{280}$ ratio to distinguish nucleic acid from protein contributions, and an LED emitting at a wavelength not absorbed by proteins (e.g., >320 nm) would allow correction for scattering. Translation from the present model-protein demonstration to a target-specific biomarker assay will therefore require integration with a molecular-recognition or separation strategy, together with a selective downstream readout. Surface-patterned ion-selective membranes have, for example, been used to develop multiplexed proteomic preconcentration devices [45]. The dual-Nafion ICP module should consequently be viewed as a preconcentration front end and an in situ monitor of the enrichment process, rather than a standalone biomarker-identification assay. In addition, the absolute magnitude of the on-chip $A_{280}$ response exceeds that predicted from the Beer–Lambert law for the 40 μm channel height (Section 4.2). Although all quantitative results were derived from the empirical on-chip calibration, independent validation of the optical response, for example by comparison with cuvette-based measurements of the same BSA standards and characterization of stray light at the aperture, will be required to establish the physical origin of this system-specific response.

The reproducibility evidence in the present study is strongest for the 10 pM condition, for which the 30 V BSA experiment, 0 V BSA control, and 30 V PBS blank were each measured using three independently fabricated chips. In contrast, the enrichment time courses at 1 µM, 10 nM, and 1 nM were presented as representative single-chip measurements; consequently, run-to-run uncertainty could not be assigned to the corresponding ${EF}_{app}$ values. A more comprehensive assessment should include replicate measurements using at least three independently fabricated chips at every initial concentration, with ${EF}_{app}$ reported as mean ± SD, quantitative run-to-run variability, device-to-device variation, in situ verification of the A_280_-to-concentration conversion, recovery experiments, and stability of the dual-Nafion confinement during repeated loading and extended operation. In situ verification could compare the $A_{280}$ response with an orthogonal measurement of the enriched plug, such as fluorescently labeled BSA, or calibrate with BSA standards prepared in buffers representative of the enrichment zone. In recovery experiments, the enriched plug would be collected and quantified using an independent protein assay. These measurements would also clarify whether protein conformational changes, aggregation, or buffer variations in the ICP zone affect the conversion from A_280_ to apparent concentration. Such data will be needed to define analytical precision and operational robustness for future translational use.

The immediate priorities for the next phase of development are therefore validation in complex biological and environmental matrices, integration of target-specific recognition elements, and systematic quantification of analytical reproducibility, recovery, and long-term device stability. Optimization of sample preparation, buffer exchange, and electrical operating conditions may also be required for higher-conductivity matrices. These directions build on the integrated dual-Nafion ICP and UV-LED optical framework reported here, but they are necessary before performance in field, clinical, environmental, food-safety, or aquaculture applications can be established.

## 5. Conclusions

A portable, integrated microfluidic platform combining dual-Nafion ion concentration polarization (ICP) preconcentration, a UV-transparent quartz substrate, a chromium-coated slot collimator, a 280 nm UV-LED, and a micro-spectrometer was developed for label-free monitoring of native BSA at 280 nm. The dual-Nafion configuration confined the enrichment zone within a fixed inter-membrane optical window, enabling time-resolved absorbance measurements during electrokinetic operation. Static BSA loading produced a linear $A_{280}$-concentration relationship over 0–60 µM (R^2^ = 0.9933) and a static-loading limit of detection of 0.91 µM. Under a 30 V bias in 0.3 mM PBS, the platform generated ${EF}_{app}$ values of approximately 41, 4.5 × 10^3^, and 1.6 × 10^4^ for initial BSA concentrations of 1 µM, 10 nM, and 1 nM, respectively. At an initial concentration of 10 pM, a reproducible, voltage-dependent increase in $A_{280}$ was detected (*n* = 3), whereas the corresponding 0 V BSA control and 30 V PBS blank remained below the detection threshold over 60 min; based on mass balance, this response was reported as qualitative detection with an enrichment-factor upper bound of 9.4 × 10^4^. The ${EF}_{app}$ values obtained with the UV-LED were of the same order of magnitude as those previously obtained with a deuterium-lamp system using the same chip design, consistent with enrichment being governed by the dual-Nafion chip rather than by the light source.

These findings demonstrate the feasibility of integrating stable dual-Nafion ICP confinement with compact, label-free UV absorbance monitoring for trace protein preconcentration in a controlled buffer system. The UV-LED provided a compact, wavelength-targeted light source for the present optical configuration without changing the underlying electrokinetic preconcentration mechanism. The platform should therefore be regarded as an upstream, label-free preconcentration and monitoring module rather than a standalone target-specific biomarker assay. Translation to clinical, environmental, food-safety, or aquaculture workflows will require validation in complex matrices, assessment of device-to-device reproducibility and analyte recovery, and integration with a target-specific molecular-recognition or separation strategy.

## Data Availability

The data presented in this study are available on request from the corresponding author.
